# Investigation of the Effect of KIR–HLA Pairs on Hepatocellular Carcinoma in Hepatitis C Virus Cirrhotic Patients

**DOI:** 10.3390/cancers13133267

**Published:** 2021-06-29

**Authors:** Takeji Umemura, Satoru Joshita, Hiromi Saito, Shun-ichi Wakabayashi, Hiroyuki Kobayashi, Yuki Yamashita, Ayumi Sugiura, Tomoo Yamazaki, Masao Ota

**Affiliations:** 1 Department of Medicine, Division of Gastroenterology and Hepatology, Shinshu University School of Medicine, Matsumoto 390-8621, Nagano, Japan; joshita@shinshu-u.ac.jp (S.J.); hiropon@shinshu-u.ac.jp (H.S.); 20hm144j@shinshu-u.ac.jp (S.-i.W.); 20hm116c@shinshu-u.ac.jp (H.K.); yukiyama@shinshu-u.ac.jp (Y.Y.); asugiura@shinshu-u.ac.jp (A.S.); ymzktm6@shinshu-u.ac.jp (T.Y.); otamasao@shinshu-u.ac.jp (M.O.); 2 Consultation Center for Liver Diseases, Shinshu University Hospital, Matsumoto 390-8621, Nagano, Japan; 3 Department of Life Innovation, Shinshu University, Matsumoto 390-8621, Nagano, Japan

**Keywords:** cirrhosis, hepatitis C virus, hepatocellular carcinoma, human leukocyte antigen, killer cell immunoglobulin-like receptors, natural killer cells

## Abstract

**Simple Summary:**

Natural killer (NK) cells normally respond to tumor cells and virally infected cells by killing them via the innate immune system. However, the functional impairment of NK cells has been observed in hepatocellular carcinoma. The NK-cell phenotype is partially mediated through the binding of killer cell immunoglobulin-like receptors (KIR) with human leukocyte antigen (HLA) class I ligands. This study evaluated the involvement of KIR–HLA pairs in hepatocellular carcinoma development in 211 patients with hepatitis C virus-associated cirrhosis. HLA-Bw4 and the KIR3DL1+HLA-Bw4 pair were significantly associated with hepatocellular carcinoma onset during a median follow-up of 6.6 years, which suggested that functional interactions between KIR and HLA or HLA-Bw4 may influence the risk of cancer development.

**Abstract:**

Natural killer cells are partially mediated through the binding of killer cell immunoglobulin-like receptors (KIR) with human leukocyte antigen (HLA) class I ligands. This investigation examined the risk of hepatocellular carcinoma (HCC) in relation to KIR–HLA pairs in patients with compensated hepatitis C virus (HCV)-associated cirrhosis. A total of 211 Japanese compensated HCV cirrhotic cases were retrospectively enrolled. After KIR, HLA-A, HLA-Bw, and HLA-C typing, associations between HLA, KIR, and KIR–HLA combinations and HCC development were evaluated using the Cox proportional hazards model with the stepwise method. During a median follow-up period of 6.6 years, 69.7% of patients exhibited HCC. The proportions of HLA-Bw4 and the KIR3DL1 + HLA-Bw4 pair were significantly higher in patients with HCC than in those without (78.9% vs. 64.1%; odds ratio (OR)—2.10, 95% confidence interval (CI)—1.10–4.01; *p* = 0.023 and 76.2% vs. 60.9%, odds ratio—2.05, *p* = 0.024, respectively). Multivariate analysis revealed the factors of male gender (hazard ratio (HR)—1.56, 95% CI—1.12–2.17; *p* = 0.009), α-fetoprotein > 5.6 ng/mL (HR—1.56, 95% CI—1.10–2.10; *p* = 0.011), and KIR3DL1 + HLA-Bw4 (HR—1.69, 95% CI—1.15–2.48; *p* = 0.007) as independent risk factors for developing HCC. Furthermore, the cumulative incidence of HCC was significantly higher in patients with KIR3DL1 + HLA-Bw4 than in those without (log-rank test; *p* = 0.013). The above findings suggest KIR3DL1 + HLA-Bw4, in addition to HLA-Bw4, as a novel KIR–HLA pair possibly associated with HCC development in HCV cirrhosis. HCV-associated cirrhotic patients with the risk factors of male gender, α-fetoprotein > 5.6 ng/mL, and KIR3DL1 + HLA-Bw4 may require careful surveillance for HCC onset.

## 1. Introduction

Chronic hepatitis C virus (HCV) infection is one of the major causes of cirrhosis and hepatocellular carcinoma (HCC) [1,2], the latter of which is prominently involved in cancer-related death [3,4]. In Japan, HCV-related HCC accounts for approximately 60% of all HCC cases [5]. The mechanisms related to the progression of HCV infection to HCC include viral, environmental, and host genetic factors [6,7].

Natural killer (NK) cells are innate lymphocytes that are crucial for the early control of several pathogenic infections and malignancies [8]. NK cells express a wide repertoire of activating and inhibitory killer cell immunoglobulin-like receptors (KIR), which detect the expression levels of major histocompatibility complex class I ligands on normal and diseased cells in humans [9]. The expression of cognate human leukocyte antigen (HLA) class I molecules on target cells results in the inhibition of NK cell-mediated cytolysis via inhibitory KIR molecules, whereas the absence or aberrant presentation of these molecules leads to the activation of NK cells via KIR and other activating receptors [10]. KIR2DL1 recognizes HLA-C group 2 (HLA-C2) allotypes that share lysine at position 80, while KIR2DL2 and KIR2DL3 are specific for HLA-C group 1 (HLA-C1) allotypes having asparagine at position 80 [11]. KIR3DL1 binds with higher affinity to HLA-Bw4 molecules containing isoleucine at position 80 (Bw4-80I) than to HLA-Bw4 molecules with threonine at position 80 (Bw4-80T) [12]. Similar to HLA molecules, KIR is highly polymorphic, which profoundly influences KIR–HLA interactions. These structural complexities, as well as extensive gene homology, have resulted in poor coverage using genome-wide association study methods, making KIR understudied as a cancer susceptibility trait.

Gene content variability at the KIR locus has been investigated as a susceptibility trait in a variety of infectious and autoimmune diseases [10]. The interactions between independently segregating KIR and HLA genes on the biologic function of NK cells have also been described for melanoma [13,14]. We previously examined the role of KIR–HLA in HCV-related HCC in a cross-sectional study and revealed that KIR2DL1 + HLA-C1 was associated with younger onset (<65 years old) HCV-related HCC [15]. Other cross-sectional studies have demonstrated relationships between KIR–HLA and HCV-related HCC [16,17], although none have addressed the development of HCC in HCV patients over long-term follow-up. As cirrhosis is one the strongest risk factors for HCC development [2], the present study investigated the risk of HCC based on KIR–HLA pairs in patients with compensated HCV-associated cirrhosis.

## 2. Results

### 2.1. Patient Characteristics

The cohort’s characteristics are summarized in Table 1. Median age was 64 years and 52% of subjects were male. The median follow-up period was 6.6 years (interquartile range: 4.1–10.0). HCV genotype 1 was detected in 180 (85.3%) patients and genotype 2 was identified in 22 (14.7%) patients. The Child–Pugh–Turcotte score was A in all subjects. Although 94 patients (44.5%) had been treated with interferon-based therapy, none achieved a sustained virological response (SVR). During the observation period, 147 (69.7%) of 211 patients exhibited HCC, with a 5-year cumulative incidence of 26.3% and a 10-year cumulative incidence of 69.7%. The HCC group had a significantly higher male ratio as compared with the non-HCC group. The α-fetoprotein (AFP) was significantly higher, while albumin, platelet count, and PT% were significantly lower in patients with HCC than in those without.

### 2.2. HLA and KIR Genotyping in Patients with HCV-Induced Cirrhosis

To clarify the impact of HLA–KIR pairs on HCC development, HLA-Bw and HLA-C were tested and KIR genes were genotyped, and their frequencies were compared between patients with and without HCC. The HLA-Bw4-positive rate was significantly higher in the HCC group than in the non-HCC group (78.9% vs. 64.1%; odds ratio (OR)—2.10, 95% confidence interval (CI)—1.10–4.01; *p* = 0.023). There were no remarkable differences between the groups for the frequencies of HLA-Bw6, HLA-C1, HLA-C2, or 14 KIR genes. HLA-Bw4 is segregated into Bw4-80I or Bw4-80T subtypes based on a dimorphism (isoleucine vs. threonine) at position 80. We observed a significant difference in the frequency of Bw4-80I positivity (*p* = 0.048), but not Bw4-80T positivity (*p* = 0.698), between the HCC and non-HCC groups (Table 2). No significant differences were detected for genotype A/A or genotype B/x between the groups (Table 2).

Since KIR interacts with specific HLA class I molecules, we examined KIR–HLA combinations in the HCC and non-HCC groups (Table 3). The frequency of KIR3DL1 + HLA-Bw4 in the HCC group was 76.2% and significantly higher than the 60.9% observed in the non-HCC group (OR—2.05, 95% CI—1.09–3.85; *p* = 0.024, corrected *p* = 0.31). Moreover, KIR3DL1 + HLA-Bw4-80I was significantly associated with HCC (63.9% vs. 48.4%; OR—1.89, 95% CI—1.04–3.42; *p* = 0.035). No differences were detected in the frequencies of the other KIR–HLA pairs between the groups. Moreover, the frequency of genotype A/A did not differ between the HCC group and the non-HCC group according to KIR3DL1 + HLA-Bw4 positivity (60.6% vs. 39.4%; *p* = 0.181) or negativity (50.9% vs. 38.5%; *p* = 0.693).

### 2.3. Risk Factors for HCC Development in HCV-Induced Cirrhosis Patients

**Figure 1:**
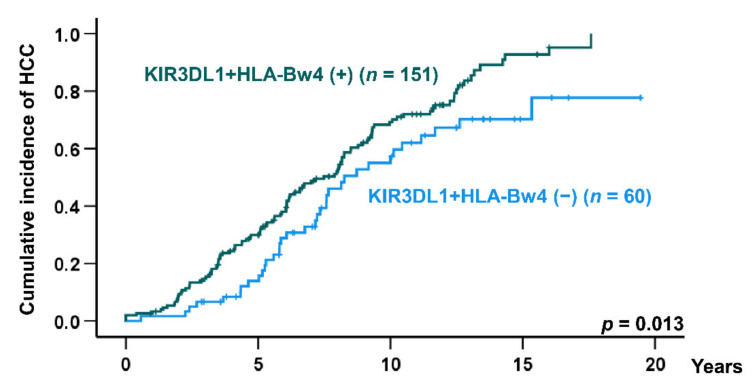
Cumulative incidence of hepatocellular carcinoma (HCC) in hepatitis C virus (HCV)-induced cirrhotic patients according to KIR3DL1 + HLA-Bw4. *p*-value was calculated by the log-rank test.

Multivariate Cox proportional hazards modeling was performed to identify risk factors for developing HCC. The AFP cut-off value was determined as 5.6 ng/mL using Youden’s index. Multivariate analysis revealed the factors of male gender (hazard ratio (HR)—1.56, 95% CI—1.12–2.17; *p* = 0.009), AFP > 5.6 ng/mL (HR—1.56, 95% CI—1.10–2.20; *p* = 0.011), and KIR3DL1 + HLA-Bw4 (HR—1.69, 95% CI—1.15–2.48; *p* = 0.007) as independent risk factors for HCC development (Table 4). Kaplan–Meier analysis showed the cumulative incidence of HCC to be significantly higher in patients with KIR3DL1 + HLA-Bw4 positivity than in KIR3DL1 + HLA-Bw4-negative patients (log-rank test; *p* = 0.013) (Figure 1).

**Figure 2:**
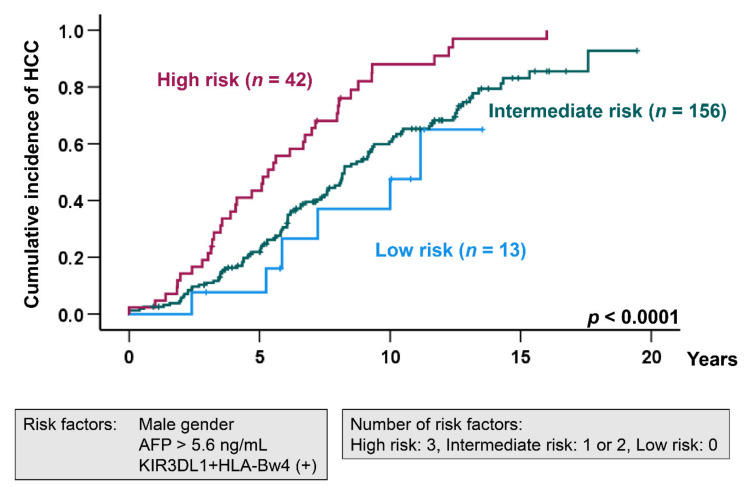
Cumulative incidence of HCC in HCV-induced cirrhotic patients according to the number of the risk factors (male gender, AFP > 5.6 ng/mL, and KIR3DL1 + HLA-Bw4). *p*-value was calculated by the log-rank test. AFP: α-fetoprotein.

We next divided the patients into three groups according to the number of risk factors identified by multivariate analysis (male, AFP > 5.6 ng/mL, and KIR3DL1 + HLA-Bw4 positive). Patients with all three risk factors were assigned to the high-risk group, those with one or two factors to the intermediate risk group, and those with no factors to the low-risk group. Kaplan–Meier testing showed that the cumulative incidence of HCC was highest in the high-risk group, followed next by the intermediate and low-risk groups (log-rank test; *p* < 0.0001) (Figure 2).

## 3. Discussion

This single-center retrospective study examined whether specific KIR–HLA pairs played a role in the development of HCC in a cohort of 211 patients with HCV-induced cirrhosis who were followed for a median of 6.6 years. Our results indicated that patients with KIR3DL1 + HLA-Bw4 had a significantly increased risk of HCC onset in Cox regression and Kaplan–Meier analyses. Since the frequency of activating KIR–HLA pairs did not differ significantly between the HCC and non-HCC groups, the inhibitory KIR3DL1+HLA-Bw4 pair, which showed a high and significantly greater frequency in the HCC group, might have contributed to HCC onset by escaping immune surveillance and other intrahepatic inflammatory processes.

Recently, Mele et al. showed that KIR haplotypes were associated with differing abilities of NK cells in HCV-infected patients [18]. Functional impairment in haplotype B carriers was evident from the presence of KIR3DL1 + HLA-Bw4 in HCV-infected subjects, and in haplotype A carriers, increased cytolytic activity was associated with higher STAT1 phosphorylation. Accordingly, we tested the frequencies of genotype A/A and genotype B/x between the HCC and non-HCC groups and in relation to KIR3DL1 + HLA-Bw4 positivity or negativity, although no significant associations were observed between genotype and KIR3DL1 + HLA-Bw4 status. Additional functional analyses of NK cells are needed to clarify the relationship between genotype and KIR3DL1 + HLA-Bw4 in HCV-associated HCC patients.

Our results showed the frequencies of HLA-Bw4 and KIR3DL1 + HLA-Bw4 in the HCC group to be 78.9% and 76.2%, respectively. Since KIR3DL1 positivity was 94.7% in HCV-associated cirrhosis in our cohort, it was difficult to conclusively ascertain HLA-Bw4 or KIR3DL1 + HLA-Bw4 as risk factors for HCC development. Moreover, Kaplan–Meier analysis showed the cumulative incidence of HCC to be significantly higher in patients with HLA-Bw4 positivity and KIR3DL1 + HLA-Bw4 positivity (log-rank test; *p* = 0.026 and *p* = 0.013, respectively). Previous studies have reported that HLA class I was associated with HCC [19,20,21,22,23]. Therefore, HLA-Bw4 may be implicated with HCC onset due to one or several HLA variants coding for molecules that affect T-cell responses [24]. In multivariate Cox regression analysis, male gender and AFP > 5.6 ng/mL were independent risk factors for the onset of HCC in addition to KIR3DL1 + HLA-Bw4 positivity, but not HLA-Bw4. The 5- and 10-year cumulative incidence rates of HCC were 43.5% and 88.0% in the high-risk group with all three factors, 21.9% and 60.7% in the intermediate-risk group with one or two factors, and 7.7% and 47.6% in the low-risk group with no factors, respectively. Thus, patients with all three risk factors tended to exhibit a more rapid progression to HCC and should be monitored more closely.

Given the functional mechanism and extensive genomic diversity of KIR and HLA ligands, specific KIR–ligand combinations have been associated with the natural clearance of HCV infection and antiviral treatment outcome in patients with chronic hepatitis C [25,26,27,28,29,30,31,32]. KIR–HLA pairs might influence disease progression as well; in our recent examination of KIR–HLA pairs in patients with autoimmune hepatitis, KIR3DL1+HLA-Bw4 was significantly related to a favorable outcome [33]. In fact, the combination of KIR3DS1 and HLA-Bw4 or HLA-Bw4-80I was under-represented in HCC patients as compared with HCV carriers without HCC in Spain and Italy [16,17]. However, our data do not confirm those results.

There are several limitations to this retrospective study. As the numbers of patients with HCC and non-HCC were too small for a definitive conclusion, a larger validation analysis with more subjects is needed to validate our results. Recent direct-acting antiviral therapies have achieved an SVR rate greater than 95% in patients with chronic hepatitis C [34,35]. Although such treatments reduce the risk of HCC development, post-SVR patients remain at an elevated risk of HCC. Since multiple genetic factors have been associated with HCC after an SVR [36,37], additional study is needed to assess whether KIR–HLA pairs are associated with HCC development after an SVR. Furthermore, HLA and KIR are very diverse and complex genes. It will be necessary to analyze those at the allele level using the latest next-generation sequencing methods. Although this study did not employ such techniques and thus could not preclude the possibility of false positives and negatives, the results of 100 control cases were consistent with those measured by the PCR–SSO method. Future studies that include allele typing are being planned. Lastly, KIR3DL1 polymorphisms are known to modulate NK-cell effector function, and the interactions of *3DL1* polymorphisms with their cognate ligands differ in their capacity to inhibit NK cell activity. This inhibitory potential is increased with the level and frequency of 3DL1 expression-encoded high-expression allotypes (*KIR3DL1-h*), low-expression allotypes (*KIR3DL1-l*), and no cell surface expression (*KIR3DL1-n*) [38,39,40]. The allotypic differences in the frequency and level of KIR3DL1 expression in affecting NK cell function were not investigated in this study. The next step is to explore the allelic level of KIR3DL1 in the development of HCC in HCV-induced cirrhotic patients.

## 4. Materials and Methods

### 4.1. Study Population and HCC Surveillance

We considered all patients consecutively referred to Shinshu University Hospital for the diagnosis and management of cirrhosis between January 1991 and December 2013 and who met the following criteria: (1) chronic HCV infection defined by positive serum HCV RNA; (2) cirrhosis proven histologically (*n* = 165) or by imaging studies (*n* = 53); (3) no infection of hepatitis B virus or human immunodeficiency virus; (4) absence of other chronic liver disease; (5) Japanese origin; and (6) no evidence of HCC at enrollment or past history of HCC treatment. After the exclusion of 7 patients who exhibited HCC within 1 year after entry, a total of 211 HCV-induced cirrhotic patients were retrospectively included in this study.

HCC surveillance was performed by examining AFP and/or des-γ-carboxy prothrombin, abdominal ultrasonography, and computed tomography and/or magnetic resonance imaging every 6 months. The diagnosis of HCC was based on imaging characteristics, arterial hypervascularity, and venous or delayed phase washout by contrast-enhanced dynamic computed tomography and/or magnetic resonance imaging when a nodular lesion was detected by ultrasonography or a tumor marker was elevated.

### 4.2. HLA Class I and KIR Typing

Whole-genomic DNA was extracted from whole-blood samples from all participants using QuickGene-800 assays (Fujifilm, Tokyo, Japan). HLA-A typing was carried out using a Luminex multi-analyzer profiling system with a LABType SSO typing kit (One Lambda, Inc., Canoga Park, CA, USA). Since HLA-A*23:01, HLA-A*24:02, and HLA-A*32:01 are known to be part of the HLA-Bw4 family, HLA-A was measured in this study. As reported in a Japanese population database, only HLA-A*24;02 was found in this study, with 23 or 32 not detected. HLA-Bw4, HLA-C1, and HLA-C2 [41] as well as KIR genes [42] were typed using the polymerase chain reaction with sequence-specific primers. HLA typing and KIR typing were used to stratify patients into groups according to predicted KIR–ligand interactions and binding affinities. The KIR–HLA pairs of interest were KIR2DL1+2DS1-HLA-C2, 2DL2/3/2DS2+HLA-C1, and 3DL1+HLA-Bw4. All testing was blinded to the clinical variables. The KIR genotype profiles were assigned to the A/A or B/x genotypes as defined previously [43].

### 4.3. Statistical Analysis

Continuous variables were compared using the Mann–Whitney U test and categorical variables were evaluated by Pearson’s chi-squared test or Fisher’s exact test, as appropriate. Receiver operating characteristic curve analysis was performed for AFP, and the area under the curve was calculated to evaluate the optimal cut-off value by Youden’s index.

The risk factors associated with the cumulative incidence of HCC were analyzed using a multivariate Cox proportional hazards model. All clinical variables included in the model were assessed at baseline. Multivariate analysis included all significant (*p* < 0.05) variables in the preceding univariate analysis. The Kaplan–Meier method and log-rank test were employed to assess the cumulative incidence of HCC. Follow-up time was censored on the date of HCC diagnosis and at the end of follow-up (31 December 2020). A *p*-value of <0.05 was considered statistically significant after Bonferroni correction for multiple testing. Statistical analyses were performed using IBM SPSS Statistics version 27 software (IBM, Tokyo, Japan).

## 5. Conclusions

In addition to HLA-Bw4, KIR3DL1+HLA-Bw4 is a novel KIR gene and KIR ligand pair that may be associated with the development of HCC in HCV-induced cirrhosis. HCV-associated cirrhotic patients with the risk factors of male gender, AFP > 5.6 ng/mL, and KIR3DL1 + HLA-Bw4 may require careful surveillance for HCC onset.

## Figures and Tables

**Table 1:** Demographic and clinical characteristics of patients with hepatitis C virus (HCV)-induced liver cirrhosis.

Characteristic	Total (*n* = 211)	Patients with Hepatocellular Carcinoma (HCC) (*n* = 147)	Patients without HCC (*n* = 64)	*p*-Value
Age, y	64 (46–78)	63 (46–77)	65 (45–80)	0.110
Male, *n* (%)	110 (52.1)	84 (57.1)	26 (40.6)	0.027
Body Mass Index	22.3 (17.6–29.8)	22.2 (17.7–29.7)	22.6 (17.2–33.5)	0.417
Albumin, g/dL	3.8 (3.0–4.5)	3.8 (2.9–4.5)	4.1 (3.1–4.5)	0.009
Bilirubin, mg/dL	0.9 (0.5–1.8)	0.9 (0.5–1.8)	0.9 (0.4–1.9)	0.532
ALT, IU/L	55 (20–200)	56 (21–209)	54 (17–201)	0.177
Platelet count, ×10^9^/L	10.6 (4.8–21.6)	10.2 (4.8–21.1)	12.0 (5.0–22.4)	0.033
Prothrombin time %	84.7 (62.3–108.0)	84.7 (58.4–104.9)	84.8 (67.9–113.1)	0.031
α-fetoprotein, ng/mL	6.9 (1.5–41.8)	9.2 (1.8–44.3)	3.7 (1.2–30.8)	<0.001
Des-gamma-carboxy prothrombin, mAU/mL	18.0 (9.5–40.0)	19.0 (9.2–40.9)	13.0 (8.2–37.6)	0.015
HCV RNA, log IU/mL	6.5 (3.4–7.3)	6.5 (3.3–7.3)	6.4 (4.0–7.4)	0.838
HCV genotype 1, *n* (%)	180 (85.3)	125 (85.0)	55 (85.9)	0.865
Interferon treatment, *n* (%)	94 (44.5)	67 (45.6)	27 (42.2)	0.649

Parameters are presented as the median (5th–95th percentiles) for continuous variables and the total number (%) for categorical variables.

**Table 2:** Frequency of HLA-Bw4, -Bw6, -C1, -C2, killer cell immunoglobulin-like receptor (KIR) genes, and KIR genotypes in patients with and without hepatocellular carcinoma (HCC).

Genetic Factor	Patients with HCC (*n* = 147)	Patients without HCC (*n* = 64)	Odds Ratio	95% Confidence Interval	*p*-Value
HLA-Bw4	116 (78.9)	41 (64.1)	2.10	1.10–4.01	0.023
HLA-Bw4-80I	97 (66.0)	33 (51.6)	1.82	1.00–3.31	0.048
HLA-Bw4-80T	31 (21.1)	12 (18.8)	1.16	0.55–2.43	0.698
HLA-Bw6	132 (89.8)	56 (87.5)	1.26	0.50–3.13	0.623
HLA-C1	145 (98.6)	64 (100.0)	-	-	0.869
HLA-C2	19 (12.9)	8 (12.5)	1.04	0.43–2.51	0.932
KIR2DL1	147 (100.0)	64 (100.0)	-	-	-
KIR2DL2	25 (17.0)	7 (10.9)	1.67	0.68–4.08	0.259
KIR2DL3	147 (100.0)	64 (100.0)	-	-	-
KIR2DL4	147 (100.0)	64 (100.0)	-	-	-
KIR2DL5	58 (39.5)	29 (45.3)	0.79	0.43–1.42	0.427
KIR2DS1	61 (41.5)	28 (43.8)	0.91	0.50–1.65	0.761
KIR2DS2	23 (15.6)	8 (12.5)	1.30	0.55–3.08	0.553
KIR2DS3	20 (13.6)	12 (18.8)	0.68	0.31–1.50	0.338
KIR2DS4	138 (93.9)	58 (90.6)	1.59	0.54–4.66	0.398
KIR2DS5	41 (27.9)	19 (29.7)	0.92	0.48–1.75	0.790
KIR3DL1	138 (93.9)	61 (95.3)	0.75	0.20–2.88	0.679
KIR3DL2	147 (100.0)	64 (100.0)	-	-	-
KIR3DL3	147 (100.0)	64 (100.0)	-	-	-
KIR3DS1	60 (40.8)	29 (45.3)	0.83	0.46–1.50	0.543
Genotype A/A	72 (49.0)	27 (42.2)	1.32	0.73–2.38	0.363
Genotype B/x	75 (51.0)	37 (57.8)	0.76	0.42–1.37	0.363

Parameters are presented as the total number (%).

**Table 3:** Frequency of killer cell immunoglobulin-like receptor (KIR)–HLA pairs in patients with and without hepatocellular carcinoma (HCC).

Genetic Factor	Patients with HCC (*n* = 147)	Patients without HCC (*n* = 64)	Odds Ratio	95% Confidence Interval	*p*-Value
KIR2DL1 + HLA-C2	19 (12.9)	8 (12.5)	1.04	0.43–2.51	0.932
KIR2DS1 + HLA-C2	9 (6.1)	5 (7.8)	0.77	0.25–2.39	0.650
KIR2DL2 + HLA-C1	24 (16.3)	7 (10.9)	1.59	0.65–3.90	0.309
KIR2DL3 + HLA-C1	145 (98.6)	64 (100.0)	-	-	0.869
KIR2DS2 + HLA-C1	22 (15.0)	8 (12.5)	1.23	0.52–2.94	0.637
KIR3DL1 + HLA-Bw4	112 (76.2)	39 (60.9)	2.05	1.09–3.85	0.024
KIR3DL1 + HLA-Bw4-80I	94 (63.9)	31 (48.4)	1.89	1.04–3.42	0.035
KIR3DL1 + HLA-Bw4-80T	30 (20.4)	12 (18.8)	1.11	0.53–2.34	0.782

Parameters are presented as the total number (%).

**Table 4:** Factors associated with HCC development in patients with cirrhosis.

Factor	HR	95% CI	*p*-Value
Sex, female vs. male	1.56	1.12–2.17	0.009
AFP (ng/mL), ≤5.6 vs. > 5.6	1.56	1.10–2.20	0.011
KIR3DL1 + HLA-Bw4, negative vs. positive	1.69	1.15–2.48	0.007

Cox proportional hazards model.

## Data Availability

The datasets used in this study are available from the corresponding author upon a reasonable request.
